# A new semi-slug of the genus *Microparmarion* from Brunei, discovered, described and DNA-barcoded on citizen-science 'taxon expeditions' (Gastropoda, Stylommatophora, Ariophantidae)

**DOI:** 10.3897/BDJ.11.e101579

**Published:** 2023-04-10

**Authors:** Menno Schilthuizen, Simon Berenyi, Nurilya Syamimi Muhammad Nazirul Ezzwan, Nur Izzah Amal Afiqah Hamdani, Harrison Wu, Luca De Antoni, Leonardo Vincenzi, Werner de Gier, Anthonie D. P. van Peursen, Iva Njunjić, Massimo Delledonne, Ferry Slik, Ulmar Grafe, Daniele Cicuzza

**Affiliations:** 1 Taxon Expeditions B. V., Leiden, Netherlands Taxon Expeditions B. V. Leiden Netherlands; 2 Naturalis Biodiversity Center, Leiden, Netherlands Naturalis Biodiversity Center Leiden Netherlands; 3 Institute Biology Leiden, Leiden University, Leiden, Netherlands Institute Biology Leiden, Leiden University Leiden Netherlands; 4 Universiti Brunei Darussalam, Bandar Seri Begawan, Brunei Universiti Brunei Darussalam Bandar Seri Begawan Brunei; 5 Taxon Expeditions, Leiden, Netherlands Taxon Expeditions Leiden Netherlands; 6 University of British Columbia, Kelowna, Canada University of British Columbia Kelowna Canada; 7 Department of Biotechnology, University of Verona, Verona, Italy Department of Biotechnology, University of Verona Verona Italy; 8 Groningen Institute for Evolutionary Life Sciences, University of Groningen, Groningen, Netherlands Groningen Institute for Evolutionary Life Sciences, University of Groningen Groningen Netherlands; 9 Environmental and Life Sciences, Faculty of Science, Universiti Brunei Darussalam, Bandar Seri Begawan, Brunei Environmental and Life Sciences, Faculty of Science, Universiti Brunei Darussalam Bandar Seri Begawan Brunei

**Keywords:** taxonomy, malacology, Borneo, new species, land snails, semi-slugs

## Abstract

**Background:**

During citizen-science expeditions to the Ulu Temburong National Park, Brunei, several individuals were collected of a semi-slug species of the genus *Microparmarion* that, based on morphology and in-the-field DNA-barcoding, was found to be an undescribed species.

**New information:**

In this paper, we describe *Microparmarionsallehi* Wu, Ezzwan & Hamdani, n. sp., after field centre supervisor Md Salleh Abdullah Bat. We provide details on the external and internal reproductive morphology, the shell and the ecology of the type locality, as well as a diagnosis comparing it with related species. DNA barcodes were generated for five individuals and used for a phylogenetic reconstruction. *Microparmarionsallehi* sp. n. and *M.exquadratus* Schilthuizen et al., 2019 so far are the only Bornean species of the genus that live in lowland forest; other species are found in montane forests.

## Introduction

Surveys of terrestrial gastropod faunas in Southeast Asia largely rely on the collection of empty shells, especially in non-calcareous regions, where densities of live snails can be very low, compared to empty shells that can be extracted from soil samples by visual search and by flotation (Schilthuizen 2011). Due to this tradition, slugs and semi-slugs have been undersampled, as they either have no shell (e.g. Philomycidae, Rathouisiidae, Veronicellidae) or a shell that will not float during flotation (Ariophantidae). Consequently, our knowledge of Southeast Asian slugs and semi-slugs almost entirely depends on live collections, usually at night, as the animals are predominantly nocturnal. Further complications to the study of these groups lie in the rarity of most species and the fact that dissection is usually required to obtain diagnostic characters. Consequently, the number of specimens in collections is low and many new species probably remain to be discovered.

During three citizen-science expeditions/field courses in the lowland forests of Ulu Temburong National Park, Brunei Darussalam, in the northwest of the island of Borneo, we found several individuals of a *Microparmarion* species. Although the species externally resembled other Bornean lowland congenerics, DNA-barcoding performed in the field lab showed that the Brunei specimens belong to a distantly-related species. Further morphological examination in the field lab confirmed this. Consequently, the material was described as a new species in a field course project.

## Materials and methods

### Fieldwork

Three expeditions/field courses were held in the Kuala Belalong Field Studies Centre (KBFSC), located at 4.54°N, 115.16°E, at 100 m elevation, which is managed by the Institute of Biodiversity and Environmental Research (IBER) and Universiti Brunei Darussalam (UBD) (Murrell et al. 2018). These field trips took place during the following periods: 25.09.-8.10.2018, 25.09.-4.10.2019 and 11-20.10.2022. The field centre is located in a primary lowland dipterocarp forest reserve with a surface area of ca. 50,000 ha (Goyes Vallejos et al. 2017), but it also includes some riverine sites with secondary forest. On each trip, teams consisting of local and international scientists, students and laypersons worked together on a combined programme of biodiversity training and exploration. During night surveys, semi-slugs were occasionally found.

### Specimens

When semi-slugs were encountered in the field, they were placed alive in plastic vials and, in the field lab, were drowned in water for a maximum of 24 hours. Each specimen received a field code. Then, in most cases, a tissue sample was removed (usually from the tail) and placed into 100% ethanol, while the rest of the body was preserved in 70% ethanol. Overall, five individuals were found and studied. Full details of the specimens are given in the Types section of the taxon treatment below. After study, specimens were deposited in the Universiti Brunei Darusaalam Museum (UBDM), Naturalis Biodiversity Center (RMNH) and the Taxon Expeditions collection (TXEX).

### Morphology

Photographs of several of the living individuals were taken in the lab. Imaging of the preserved individuals and their shells was done with a smartphone through the eyepiece of a Nikon C-PSN dissection microscope. Pencil drawings of shells were made by tracing photos on a laptop computer screen. Descriptions of the external morphology were made by careful examination of specimens maintained in 70% ethanol. Dissections were carried out in 70% ethanol, using two pairs of sharp dissection forceps and imaged by fixing the dissected parts on a black wax surface with insect pins, submerging them in 70% ethanol, then fixing the tissue with 100% ethanol, removing the pins and photographing with a smartphone through the eyepiece of the dissection microscope. Even though the internal structure of the male genitalia may contain relevant diagnostic features, we refrained from opening the penis and epiphallus in the only adult specimen we had, so as not to prevent interpretation of the external form in future studies.

### Molecular work

The extraction of DNA was done by cutting small pieces of semi-slug tissue and performing the DNeasy Blood & Tissue kit (Qiagen) protocol, which yielded the template to be used during PCR, to amplify the *cytochrome c oxidase subunit I* (*COI*) barcoding region using the primers LCO1490 and HC02198 of Folmer et al. (1994), tailed at 5’ with Oxford Nanopore adapters. The quality control of the DNA quantification of the DNA concentration was done by performing a Qubit fluorometer test and testing its integrity using agarose gel. Successful bands allowed the purification of the samples, to avoid contaminants, using AMPure XP Beads. Every amplicon was marked with a specific barcode using the PCR-barcoding kit (Oxford Nanopore Technologies). DNA libraries were prepared from pools of amplicons with the Ligation Sequencing Kit 1D (Oxford Nanopore Technologies). Samples were then sequenced using the MinION device (Oxford Nanopore Technologies) in an R9.4 flowcell that can operate up to 36 GB/run (Menegon et al. 2017,Maestri et al. 2019). After sequencing, the data from the MinION device were base-called using a pipeline performed by the ONT Preprocessing Pipeline software that base-calls sequences. These reads were further examined with the OnTrack2, enabling rapid and accurate taxonomic assignment. The sequences (as well as data and images of the corresponding specimens, with sample IDs as displayed in the list of material below) were deposited on the Barcoding of Life Database (BOLD; www.boldsystems.org) under numbers TXEX074-23, TXEX075-23, TXEX076-23, TXEX077-23 and TXEX078-23.

### Phylogenetic analysis

To confirm conspecificity of all specimens and monophyly of the new species and also to ascertain its status with respect to related species, we downloaded all available DNA barcodes for *Microparmarion* from BOLD and GenBank. These, together with the newly-generated sequences and four sequences of *Parmarionmartensi* Simroth, 1893, to be used as outgroup, were aligned in Geneious Prime v.2023.0.1 using the MUSCLE 5.1 (Edgar 2004) option, the PPP algorithm and otherwise default settings. The aligned 31 sequences were then phylogenetically analysed using the PhyML plug-in of Geneious Prime (Guindon et al. 2010). The evolutionary model was general time-reversible, fixed proportion of invariable sites, four substitution rate categories, estimated gamma distribution and 100 bootstrap replicates. Trees were managed in FigTree v.1.4.2 and the *P.martensi* outgroup sequences (GenBank accession number FJ481180 and BOLD record numbers KCAS058-14–KCAS060-14) were used for rooting the trees.

## Taxon treatments

### Microparmarion sallehi

Wu, Ezzwan & Hamdani sp. n.

FAE460A4-A4E2-5E4F-8C74-1603B9095381

08137C97-B73B-4E05-A915-5B56AE5048C9

#### Materials

- **Type status:** Holotype. **Occurrence:** catalogNumber: UBDM.7.00152; recordedBy: Simon Berenyi; individualCount: 1; lifeStage: adult; occurrenceID: E8F80EB3-496A-55DB-AB38-9B8F961214A7; **Taxon:** scientificName: Microparmarionsallehi; **Location:** country: Brunei; stateProvince: Temburong District; locality: Kuala Belalong Field Studies Centre, Ashton trail; verbatimElevation: 100 m; decimalLatitude: 4.547; decimalLongitude: 115.157; georeferenceProtocol: GPS; **Identification:** identifiedBy: Menno Schilthuizen; dateIdentified: 2022; **Event:** eventDate: 26/09/2018; **Record Level:** language: en; basisOfRecord: PreservedSpecimen
- **Type status:** Paratype. **Occurrence:** catalogNumber: RMNH.MOL.328207; recordedBy: Menno Schilthuizen; individualCount: 1; lifeStage: juvenile; occurrenceID: DABF18BA-CA1A-5E23-8436-C2902ACB3909; **Taxon:** scientificName: Microparmarionsallehi; **Location:** country: Brunei; stateProvince: Temburong District; locality: Ulu-Ulu Resort Helipad; verbatimElevation: 100 m; decimalLatitude: 4.555; decimalLongitude: 115.153; georeferenceProtocol: GPS; **Identification:** identifiedBy: Menno Schilthuizen; dateIdentified: 2022; **Event:** eventDate: 14/10/2022; **Record Level:** language: en; basisOfRecord: PreservedSpecimen
- **Type status:** Paratype. **Occurrence:** catalogNumber: TxEx-BR2202; recordedBy: Iva Njunjić; individualCount: 1; lifeStage: juvenile; occurrenceID: E66016F3-86E1-5F46-AC2C-6C3D1535162D; **Taxon:** scientificName: Microparmarionsallehi; **Location:** country: Brunei; stateProvince: Temburong District; locality: Kuala Belalong Field Studies Centre, Ashton trail; verbatimElevation: 100 m; decimalLatitude: 4.547; decimalLongitude: 115.157; georeferenceProtocol: GPS; **Identification:** identifiedBy: Menno Schilthuizen; dateIdentified: 2022; **Event:** eventDate: 16/10/2022; **Record Level:** language: en; basisOfRecord: PreservedSpecimen
- **Type status:** Paratype. **Occurrence:** catalogNumber: TxEx-BR2203; recordedBy: Iva Njunjić; individualCount: 1; lifeStage: juvenile; occurrenceID: 82288ED8-A035-581D-B1CC-3EC3838F2953; **Taxon:** scientificName: Microparmarionsallehi; **Location:** country: Brunei; stateProvince: Temburong District; locality: Kuala Belalong Field Studies Centre, Ashton trail; verbatimElevation: 100 m; decimalLatitude: 4.547; decimalLongitude: 115.157; georeferenceProtocol: GPS; **Identification:** identifiedBy: Menno Schilthuizen; dateIdentified: 2022; **Event:** eventDate: 16/10/2022; **Record Level:** language: en; basisOfRecord: PreservedSpecimen
- **Type status:** Other material. **Occurrence:** catalogNumber: TxEx-BR1901; recordedBy: Taxon Expeditions; individualCount: 1; lifeStage: juvenile; occurrenceID: 4B28FD4F-841C-5A38-A713-632C00FB1C1B; **Taxon:** scientificName: Microparmarionsallehi; **Location:** country: Brunei; stateProvince: Temburong District; locality: Kuala Belalong Field Studies Centre, Ashton trail; verbatimElevation: 100 m; decimalLatitude: 4.547; decimalLongitude: 115.157; georeferenceProtocol: GPS; **Identification:** identifiedBy: Menno Schilthuizen; dateIdentified: 2022; **Event:** eventDate: 30/09/2019; **Record Level:** language: en; basisOfRecord: PreservedSpecimen

#### Description

**Figure 1.**
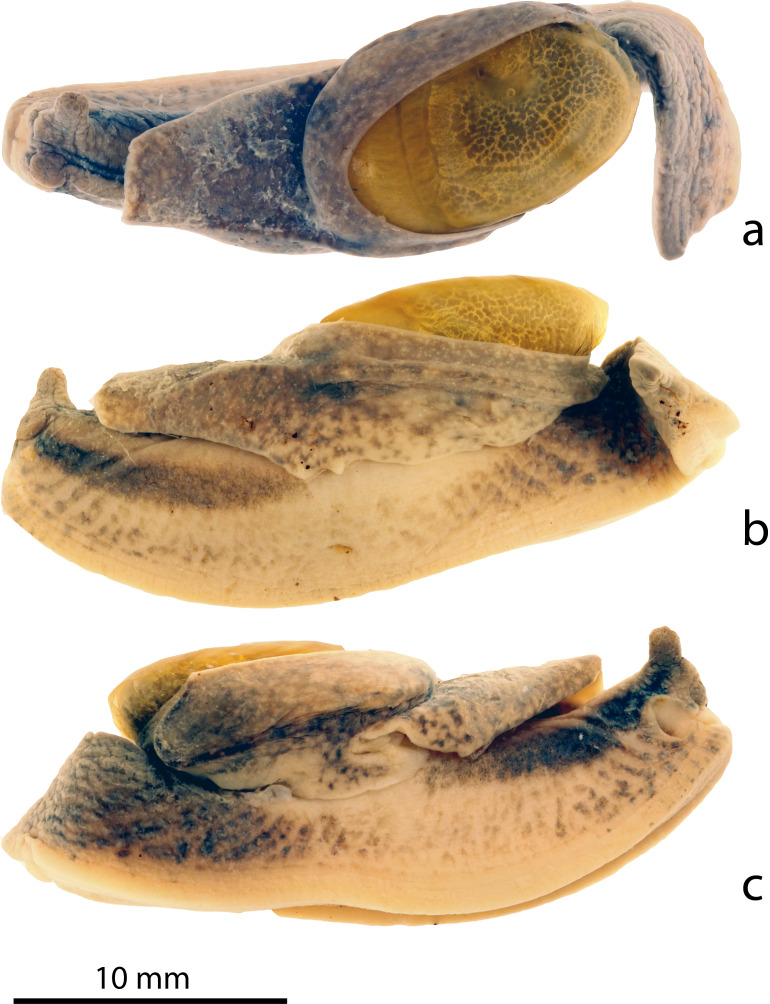
*Microparmarionsallehi*, n. sp., photographs of preserved holotype specimen (UBDM.7.00152), Kuala Belalong Field Studies Centre, Brunei Darussalam; **a** dorsal view; **b** left lateral view; **c** right lateral view.

**Figure 2.**
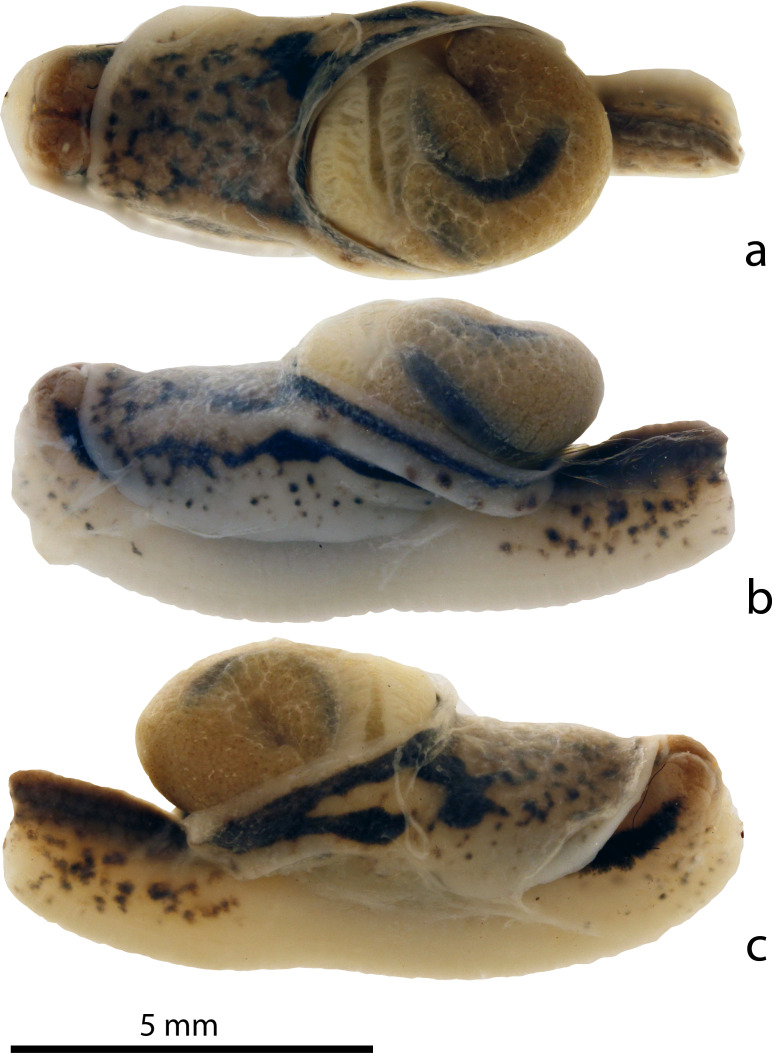
*Microparmarionsallehi*, n. sp., photographs of preserved paratype specimen (TxEx-BR2203), Kuala Belalong Field Studies Centre, Brunei Darussalam; **a** dorsal view; **b** left lateral view; **c** right lateral view.

Preserved specimens (Figs 1, 2). Ethanol-preserved juveniles are 6–11 mm in length. On the dorsal and lateral sides excluding the foot and its margins, there is a blackish-brown mottled pattern on a pale white background. The foot is pale white with a smooth surface, with the lateral head margins of the foot having a blackish-brown mottled pattern; the rest of the margins remain pale white. Three distinct longitudinal dark brown bands run on the head, one starting posterior of each eye tentacle and the third dorsomedially in between these two, starting level with them. These three bands end at the level of the pneumostome. In between the rim of the shell and when the left and right mantle lobes are pulled back, there can be either blackish-brown mottled patterns or stripes that encircle the shell. An unpigmented peripheral ridge runs from the left frontal margin of the mantle, immediately behind the left eye tentacle, circles the shell, where, on the caudolateral side of the shell, it throws off a short side branch that leads dorsad to the mantle edge; the main branch of the keel continues and ends on the right at the pneumostome. The genital opening is visible on the right lateral side behind the head and appears as a circular pore. In the adult, 29 mm long, the colour patterns are similar to those in the juveniles, but the contrasts are much reduced and colours range from pale light brown to reddish-brown.

**Figure 3.**
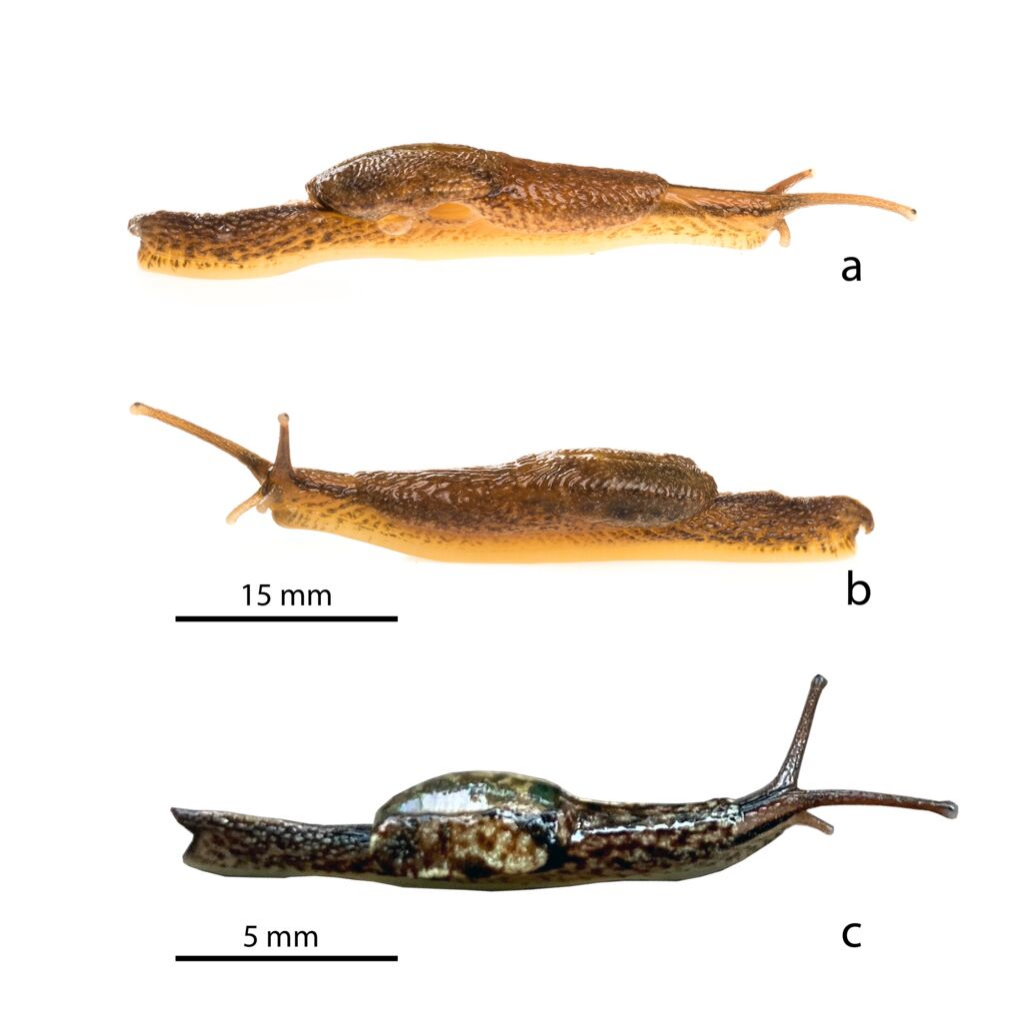
*Microparmarionsallehi*, n. sp., photographs of living individuals, Kuala Belalong Field Studies Centre, Brunei Darussalam; **a** holotype, UBDM.7.00152, right lateral view; **b** holotype, UBDM.7.00152, left lateral view; **c** paratype, TxEx-BR2201, right dorsolateral view.

Living specimens (Fig. 3). Living specimens differ from preserved ones in size (in ethanol, the animals contract to 50% of their live extended length) and in colouration, as follows. The general colouration for the dorsal side and ventral side is blackish-brown and pale white, respectively. In juveniles, the foot is pale white with a smooth surface, with the lateral frontal margins of the foot having a blackish-brown mottled pattern and the rest of the margins remaining pale white. In adults, this blackish-brown mottled pattern can not only extend to the frontal margins of the foot, but also the caudal margins. The mantle carries a blackish-brown mottled pattern on a pale background, with a yellowish-white streak extending from the left mantle lobe to the mantle itself. In adults, the pattern on the mantle largely disappears and is replaced by an ochre colouration, with the streak being reduced or becoming non-existent. The left and right mantle lobes in both adults and juveniles meet along a curved slit. Anterior to the mantle in juveniles, two longitudinal black stripes on the head that start caudally at the base of the eye tentacles extend towards the anterior part of the mantle. In addition, a dorso-medial black line on the head in between the eye tentacles also extends caudally towards the mantle. In juveniles, all four tentacles may be reddish- or greyish-brown (though the oral tentacles can be pale as well), but in the adult, this is reduced to an ochre brown, with the oral tentacles becoming paler. The caudal horn, dorsal to the caudal end of the foot, is pointed caudally and slightly extends beyond the length of the foot. In the adult, the caudal horn can be pointed or curved downwards. In juveniles, the colouration of the tail is a greyish-brown, with a dark brown mottled pattern. In the adult, this high-contrast colouration is replaced with a general browner colouration, though some mottling remains.

##### Shell

**Figure 4.**
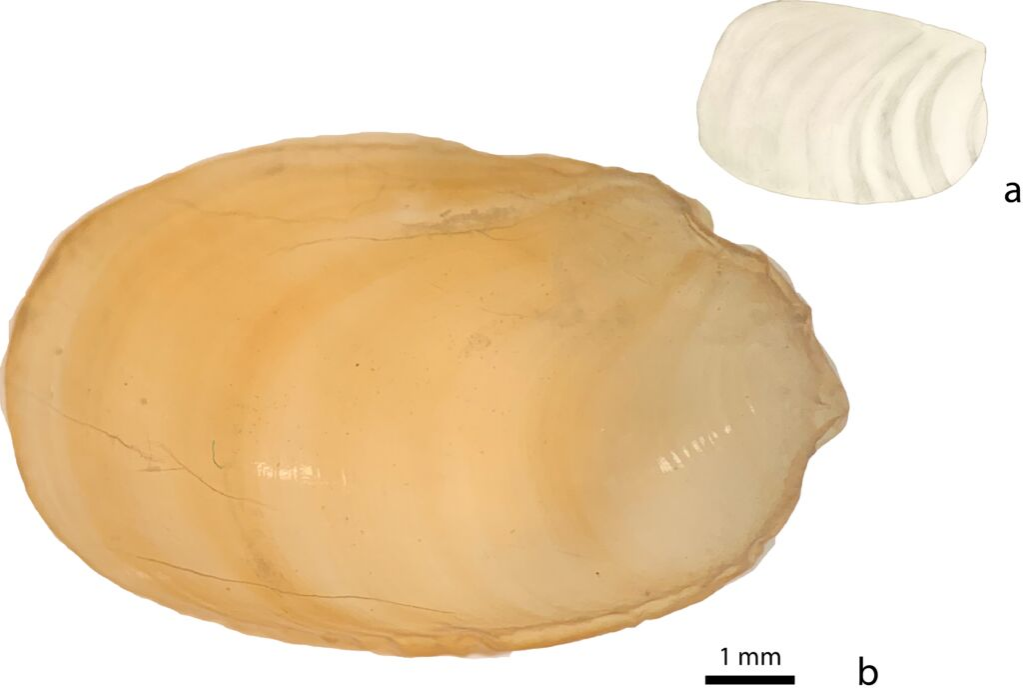
*Microparmarionsallehi*, n. sp., dorsal views of shells; **a** pencil drawing of the shell of a paratype specimen (TxEx-BR2201), Helipad, Ulu-Ulu Resort, Brunei Darussalam; **b** photograph of the shell of the holotype specimen (UBDM.7.00152), Kuala Belalong Field Studies Centre, Brunei Darussalam.

Juvenile shells (Fig. 4a) are oval, 3.5–6.0 mm long and 2.0–4.0 mm wide, of a transparent yellowish-green colouration, slanting towards the left lateral side, which causes a bulge on the left side. The surface is smooth and developmental rings are visible. In lateral view, the shell rim is flattened while the shell smoothly curves into a round bump on top. Whorls are not discernible: the shell consists of a single, oval dish. The adult shell (Fig. 4b) is 11 mm long and 8 mm wide, fingernail-like and similarly displays several darker-coloured growth bands, but no traces of whorls.

##### Genitalia

**Figure 5.**
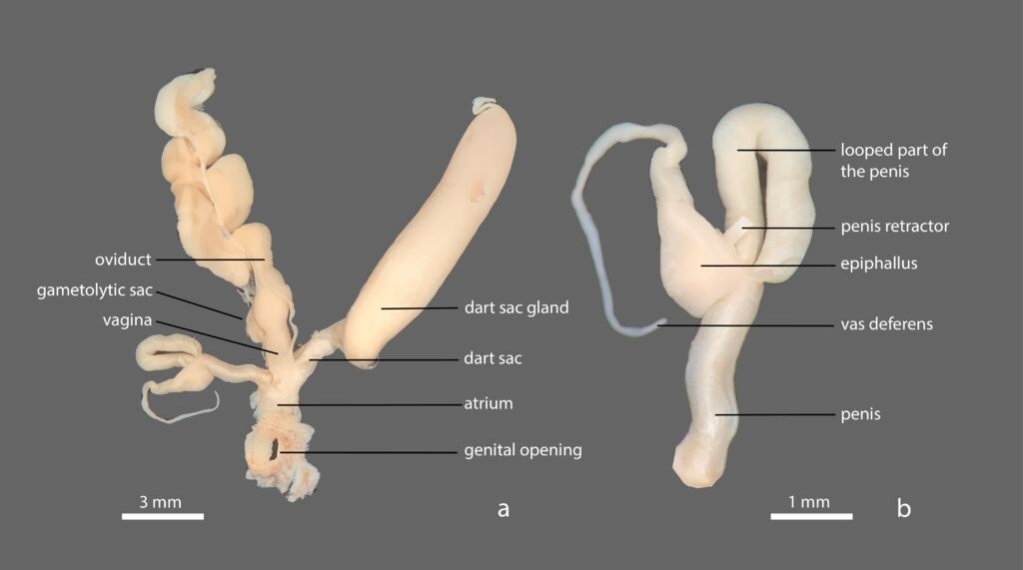
*Microparmarionsallehi*, n. sp., preserved holotype specimen (UBDM.7.00152), Kuala Belalong Field Studies Centre, Brunei Darussalam; **a** dissected genitalia; **b** detail of penis and epiphallus.

The penis, at around 5 mm from the connection with the vagina, bends back upon itself in a 2-mm-long loop (Fig. 5). The looped part is of the same width as the proximal part of the penis. Distally, the looped part narrows abruptly and is enveloped by a muscular extension of the penis; the epiphallus, 2.2 mm long, is visible as an initially relatively thick, but then narrowing, sinuous tube inside this muscular sheath. The penis retractor muscle appears to connect at the basis of the epiphallus. Terminally, the epiphallus gives way to the vas deferens. At 4 mm from the genital opening, the gametolytic sac emerges from the vagina. The dart sac is ca. 5 mm long and connects to the vagina at the opposite side from the penis. It is connected to the 10-mm-long, 2-mm-wide dart sac gland by a narrow looped stalk.

##### DNA barcode

The *COI* DNA barcode of the holotype (UBDM.7.00152; BOLD TXEX074-23) is as follows:

5’AACATTATATATAATTTTTGGAGTTTGATGTGGTATAGTAGGAACAGGCTTATCATTATTAATTCGATTAGAATTAGGTACAGCTGGTGTATTACTTGATGATCATTTTTTTAATGTAATTGTGACTGCACATGCATTTGTAATAATTTTTTTTATAGTTATACCTATTATAATTGGAGGATTTGGAAATTGAATAGTTCCTTTATTAATTGGAGCTCCAGATATAAGTTTTCCACGAATAAATAATATAAGATTTTGATTATTACCTCCTTCATTTGTATTTTTAATTGTTTCTAGTATGGTTGAAGGTGGTGCAGGAACTGGTTGAACAGTTTATCCACCTTTAAGAGGACCAGTAGGACATGCAGGAGCCTCAGTAGATTTGGCTATTTTTTCTTTACATTTAGCAGGTATATCATCTATTTTAGGTGCTATTAATTTTATTACTACTATTTTTAACATACGGTCTTCTGGTATAACTATAGAACGTGTAAGACTATTTGTTTGATCTATTTTAGTAACTGTATTTTTATTACTATTATCTTTACCTGTACTTGCTGGGGCTATTACAATACTTTTAACTGATCGAAATTTTAATACTAGTTTTTTTGATCCTGCTGGAGGAGGAGATCCGATTTTATATCAACATTTATTT3’

#### Diagnosis

This new species of *Microparmarion* stands out because of its shell, which, unlike other north Bornean species of the genus, has no discernible whorls. In that sense, it appears to be more closely related to *Parmarion*, with which it also shares the clearly keeled mantle. However, the genital set-up, with the looped part of the penis, the vas deferens connected terminally to the epiphallus and the unstalked gametolytic sac clearly fits with the *Microparmarion* genus definition (Simroth 1893; Vermeulen and Liew 2022). The looped part of the penis, which remains thick until the place where it is enveloped by the muscular sheath, places the species in ‘group 2’ of Vermeulen and Liew (2022).

Externally similar north Bornean species are *M.simrothi* Collinge & Godwin-Austen, 1895, *M.exquadratus* Schilthuizen et al., 2019, and *M.convolutus* Vermeulen & Liew, 2022. What sets *M.sallehi* apart from *M.simrothi* is that the new species lacks *M.simrothi*’s papillose mantle surface and has partial mottled patterns on the foot margins, whereas in *M.simrothi* the foot margin has an uninterrupted mottled pattern. The mantle of the new species lacks the uneven, interrupted dark ring close to the periphery of the part of the mantle that covers the shell. In adult *M.convolutus*, the mantle is smooth, while in the new species, it is covered in low nodules and ripples. The colouration in *M.convolutus* is whitish, pale orange or pale pink with a slight greyish mottling on the sides and the tail, while the new species is a pale orange as an adult or pale white as a juvenile with a blackish-brown mottled pattern. *M.exquadratus*, finally, is generally darker in colour: dull orange to pale red with dark brown patterning, while the new species is a pale orange as an adult or pale white as a juvenile with a blackish-brown mottled pattern. *M.sallehi* has either uninterrupted blackish-brown mottled patterns or horizontal stripes that encircle the shell in juveniles. In adults, these patterns or stripes do not appear. *M.exquadratus*, on the other hand, has a mantle with an uneven, wide, locally interrupted dark ring surrounding the lumen otherwise coarsely spotted. In live *M.exquadratus*, the surface of the mantle is covered in small, irregular pustules, whereas in *M.sallehi*, there are parallel ripples.

In the anatomy, *M.sallehi* n. sp. differs from all these three similar species by the long, strongly developed looped part of the penis, which is as thick and as long as the proximal part of the penis. In *M.simrothi*, *M.exquadratus* and *M.convolutus*, the looped part is always much thinner or differently shaped.

#### Etymology

The species epithet is a masculine genitive and honours the supervisor of the Kuala Belalong Field Studies Centre, Mr. Md Salleh Abdullah Bat, who recently retired. The taxon expedition, during which the species was described, was the last group he hosted before retirement. The name won the winning number of votes out of seven options during a voting session in which all participants of the 2022 expedition took part.

#### Ecology

This species is generally crepuscular and nocturnal. It was seen at night and in the morning after rain, crawling underneath leaves of saplings and on vines, in both primary and secondary forest.

## Discussion

**Figure 6.**
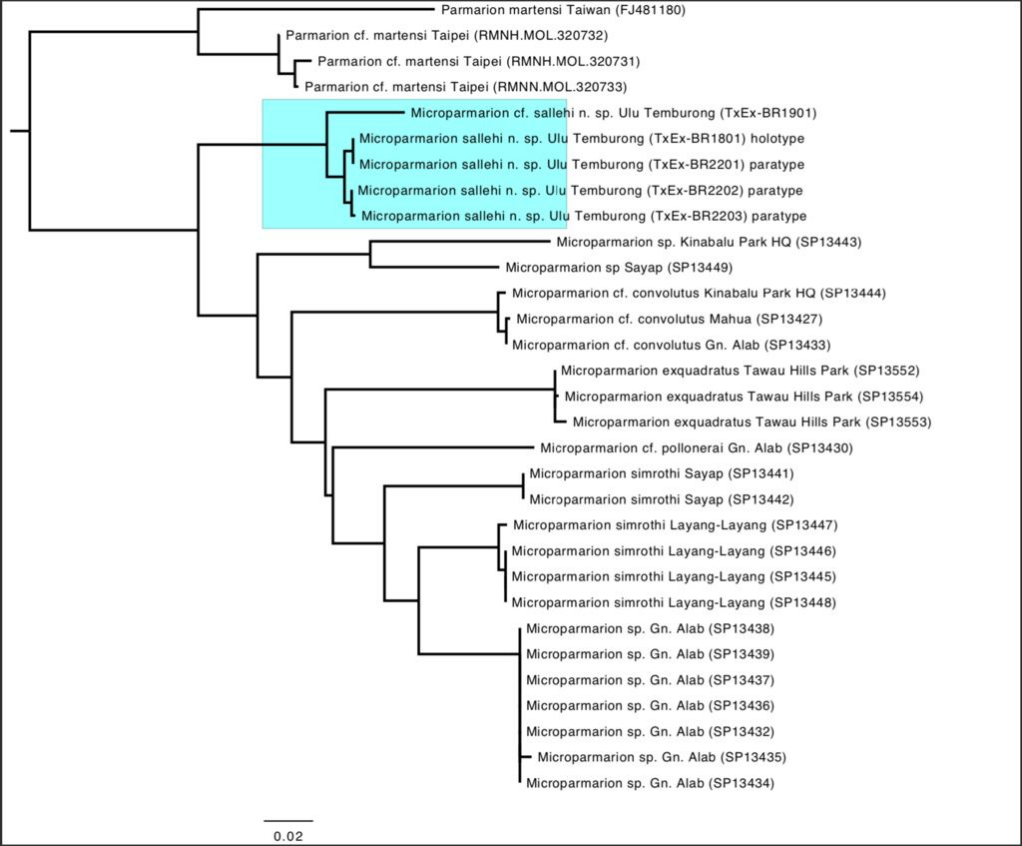
Molecular phylogenetic reconstruction for *M.sallehi* n. sp. (highlighted in blue) and other Bornean species of the genus *Microparmarion*. Numbers above branches are bootstrap values.

Our phylogenetic analysis (Fig. 6) suggests that *M.sallehi* forms a distinct, monophyletic species, but one sample, TxEx-BR1901, although morphologically identical, is genetically somewhat divergent. This may be due to artifactual autapomorphic mutations in the sequence or the species may actually belong to a different, sympatric, closely-related species. For this reason, this specimen has not been used for the description and is not part of the type series.

In northern Borneo, the genus *Microparmarion* appears to be more diverse than was suspected until recently. In an unpublished manuscript (Vermeulen, Schilthuizen & Liew, 2005, unpublished) for a guide for the land snails of Sabah, only two species were reported: *M.simrothi* Collinge & Godwin Austen, 1895 and *M.pollonerai* Collinge & Godwin Austen, 1895, both from montane forest. However, recent work has greatly augmented this number. Schilthuizen (2004) mentioned a possible species from the Crocker Range that he could not class with either of the previously-described species and Schilthuizen and Liew (2008) reported another undescribed species from Long Pa Sia. Schilthuizen et al. (2018) then described a lowland species, *M.exquadratus*, while also showing, in a phylogenetic analysis of *COI* DNA-barcodes, that at least five more undescribed species were likely to be present in northern Borneo. Vermeulen and Liew (2022) then described the species previously reported by Schilthuizen (2004) as *M.basifixus* Vermeulen & Liew, 2022 and another species from lower montane forest of the Crocker Range as *M.convolutus* Vermeulen & Liew, 2022. The present paper adds another lowland species from Brunei, which may also occur in Sarawak and/or Sabah. Therefore, the total number of species of *Microparmarion* in northern Borneo may amount to more than ten, of which at least half remain to be described.

Overall, *Microparmarion* species appear to be infrequently encountered. This may partly be due to true rarity, but also to the facts that their shells resist the commonly-used flotation techniques and that the living individuals are only seen during night or particularly wet weather. We would like to encourage malacologists working in Borneo and elsewhere in Southeast Asia to pay particular attention to these elusive semi-slugs, so that eventually sufficient material will be available for a genus-wide revision (a revision in which also related, possibly insufficiently circumscribed genera of the Ostracolethinae would need to be included).

Interestingly, *M.sallehi* appears to be the most basal branching species of the genus. The other lowland species, *M.exquadratus*, is quite distantly related and separated from *M.sallehi* by several highland species. This might suggest that speciation in the genus has repeatedly been accompanied by elevational niche shifts, as described for other taxa in Borneo (Moyle et al. 2005,Merckx et al. 2015). However, bootstrap support values are very low, so further reconstructions of the phylogeny and niche evolution will have to await greater taxon sampling and the inclusion of additional genetic loci.

## Supplementary Material

XML Treatment for Microparmarion sallehi
